# Persistent left superior vena cava detected after central venous catheter insertion

**DOI:** 10.1186/2193-1801-3-437

**Published:** 2014-08-15

**Authors:** Jan M Sohns, Martin Fasshauer, Wieland Staab, Michael Steinmetz, Christina Unterberg-Buchwald, Jan Menke, Joachim Lotz

**Affiliations:** Institute for Diagnostic and Interventional Radiology, Georg-August-University, Göttingen, Germany; German Center for Cardiovascular Research, DZHK, Göttingen, Germany; Department of Cardiology and Pneumology, Georg-August-University Göttingen, Göttingen, Germany; Department of Pediatric Cardiology and Intensive Care Medicine, Georg-August-University, Göttingen, Germany; Institute for Diagnostic and Interventional Radiology, Center of Radiology, DZHK, Georg-August-University Göttingen, UMG Universitätsmedizin Göttingen, Robert-Koch-Str. 40, 37075 Göttingen, Germany

**Keywords:** Persistent left superior vena cava, Vena cava, Venous catheter installation, Cardiac CT, Chest x-ray, Coronary sinus

## Abstract

**Introduction:**

Persistent left superior vena cava is a rare case with an appearance of 0.3% to 0.5% of individuals in general population. Indication for jugular venous intervention could be different, such as implantable venous catheters for oncological therapy. The present report describes a case of a patient with a persistent left superior vena cava detected after central venous catheter (CVC) installation using computer-assisted tomography (CT).

**Case description:**

In a control chest X-ray the CVC was not in the right superior vena cava as expected to be. A following blood gas analysis revealed venous concentration. The consultation of additional CT diagnostics yielded a persistent left superior vena cava with an outlet to dilated coronary sinus.

**Discussion and evaluation:**

The patient was followed over 1 year with the underlying disease of chronic obstructive pulmonary disease. Cardiac insufficiency, sinus aneurysm and arryhtmias could appear with growing age in patients with persistence left superior vena cava, but most of them are asymptomatic. Knowing the venous anatomy is important for correct position and function of e.g. totally implantable venous catheters, central lines or pacemakers.

**Conclusion:**

The importance of early imaging diagnosis of this anatomical variation could optimize adequate therapy and finally improve living conditions. CT can help adapting correct therapy with correct diagnostics.

## Background

A persistent left superior vena cava is a rare diagnosis with an appearance of 0.3% to 0.5% of individuals in the general population (Sarodia and Stoller 2000; Povoski and Khabiri 2011; Parreira et al. 2009; Granata et al. 2009; Goyal et al. 2008). Nevertheless it is the most common thoracic venous anomaly (Wissner et al. 2010; Jolly et al. 2010; Lee et al. 2011; Gonzalez-Juanatey et al. 2004; Tsutsui et al. 2010). Usually, the left superior vena cava disappears after embryological developement. The diagnosis can be missed by the presence of a normal right superior vena cava. This patient did not have a normal or rest of a right superior vena cava.

Most of the patients are asymptomatic and the presence of the persistent left superior vena cava is incidentally found during or after insertion of a CVC or pacemaker leads. In this patient the CVC was not in the right superior vena cava as expected to be after check up in a chest X-ray. The correct description of a persistent left superior vena cava and absence of a right superior vena cava has important clinical implications in certain situations, such as oncological therapy, totally implantable venous catheters, central lines, hemodynamic monitoring in intermediate care unit (ICU) or the correct position of pacemakers (Sarodia and Stoller 2000; Povoski and Khabiri 2011; Parreira et al. 2009; Granata et al. 2009; Goyal et al. 2008; Wissner et al. 2010; Jolly et al. 2010; Lee et al. 2011; Gonzalez-Juanatey et al. 2004; Tsutsui et al. 2010). Further clinical relevance of the described anomaly could be due to common tachyarrhythmia and conduction disturbances (Motta-Leal-Filho et al. 2014; Giebel et al. 2000; Schummer et al. 2003; Schiffmann et al. 2003; Peltier et al. 2006).

## Case description

**Figure 1:**
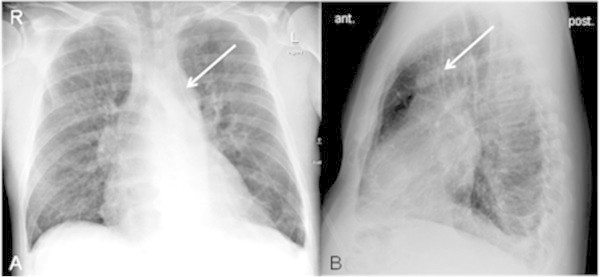
**CVC in chest X-ray. A**: Anterior to posterior chest X-ray with a CVC installation (arrow) in the persistent left superior vena cava detected (lying patient). **B**: lateral view showing the catheter (standing patient).

**Figure 2:**
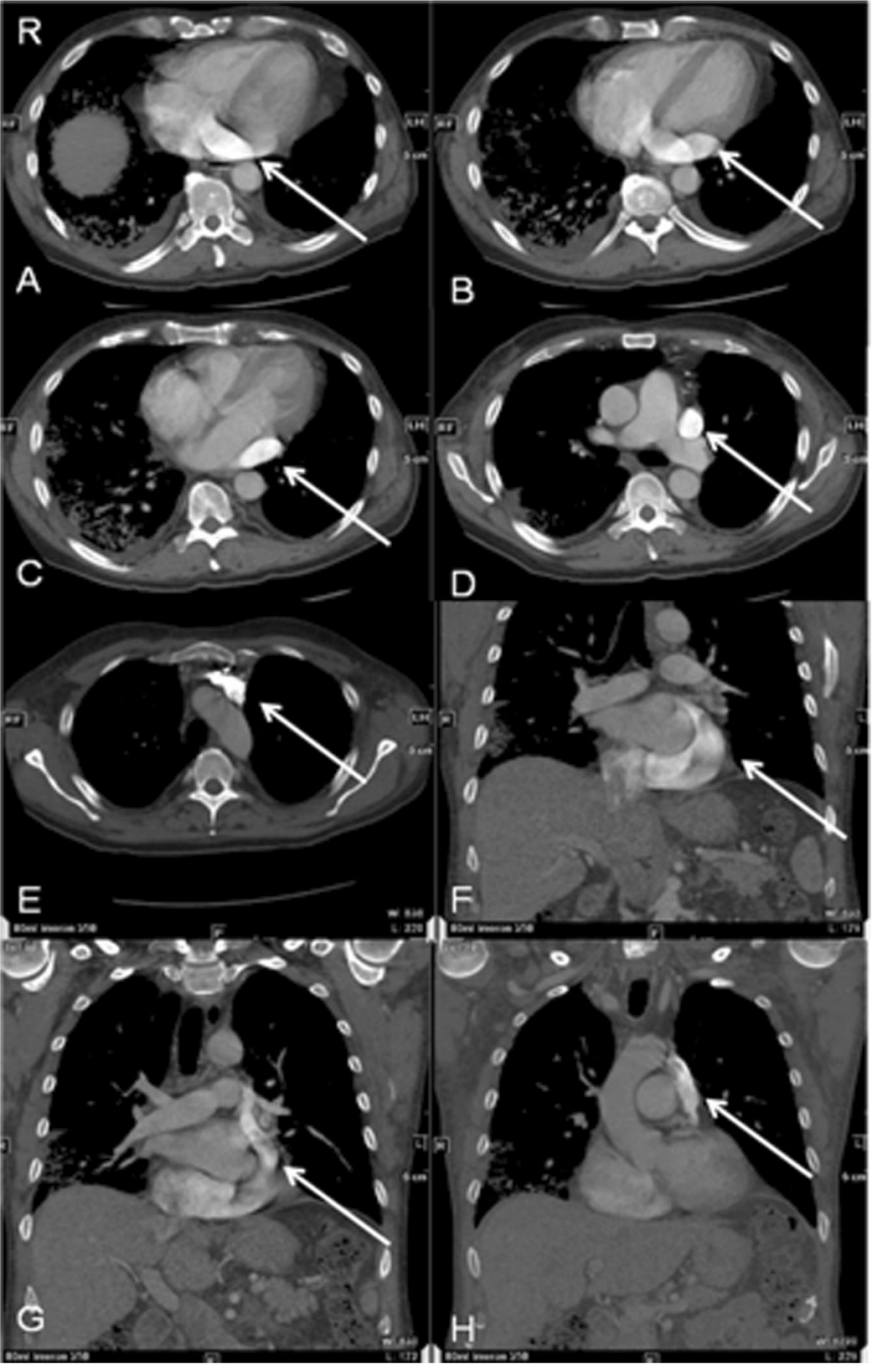
**Persistent left superior vena cava in CT. A**: Ostium of the persistent left superior vena cava (arrow) to the coronary sinus. **B-E**: Cranial-caudal rout of the persistent left superior vena cava. **F-H**: Coronal view of the persistent left superior vena cava.

**Figure 3:**
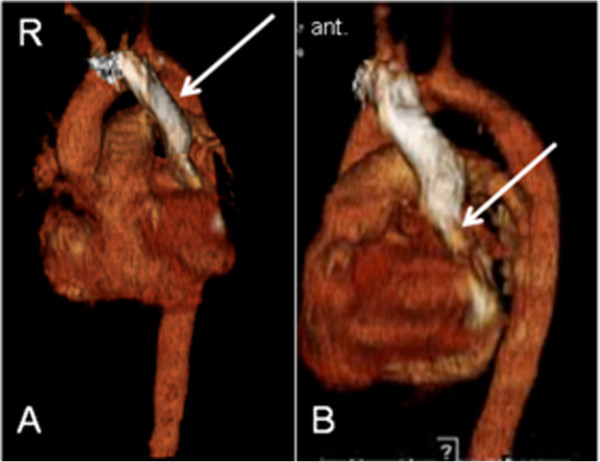
**Persistent left superior vena cava in CT in 3D-reconstruction. A**: 3D-reconstruction view from anterior left-sided showing the heart anatomy and the relation to the persistent left superior vena cava. **B**: Lateral view from the left side showing the entrance to the coronary sinus.

A 60-year old patient with acute myeloid leukemia got a CVC installation for infusion of chemotherapy in the left jugular vein. The anterior to posterior chest X-ray documented a postion of the catheter tip near the aorta (Figure 1). A following blood gas analysis revealed venous concentration. Additional computed tomography (CT) diagnostics were performed to clarify the situation. CT (64-slice VCT Light Speed, GE Healthcare, Milwaukee, USA) yielded a persistent left superior vena cava with an outlet to a dilated coronary sinus into the right atrium (Figure 2). There was no evidence for a right superior vena cava (Figure 3). Both subclavian veins and jugular veins drained into the left superior vena cava. There was no specific right-sided brachiocephalic vein. As secondary finding, the patient suffered from severe chronic obstructive pulmonary disease. Echocardiography was not performed, because it was a kind of unknown emergency situation and CT was available.

## Discussion and evaluation

This anatomical variation is important against the background of minimal invasive procedure such as totally implantable venous catheters, central lines or pacemakers.

The patient had a persistent left superior vena cava with drain of both subclavian and all head and neck veins leading to a dilated coronary sinus. The CT helped to understand the location of the CVC in detail and clearly showed venous anatomy. Persistence of the vena cava superior is the most common congenital venous anomaly of the thoracic systemic occurring in 0.3% to 0.5% of individuals in the general population, and up to 12% of individuals with other documented congenital heart abnormalities such as atrial septal defect, ventricular septal defect, aortic coarctation, transposition of the great vessels, tetralogy of Fallot, and anomalous connections of the pulmonary veins (Sarodia and Stoller 2000; Povoski and Khabiri 2011; Parreira et al. 2009; Granata et al. 2009; Goyal et al. 2008). Exact anatomy is important in patients who need a central venous line for medical therapy or who need implantation of pacemaker leads. As seen in our case, a single persistent left superior vena cava without a right superior vena cava is more rare than having both. In a previous study only three patients out of ten showed this anatomic variation (Gonzalez-Juanatey et al. 2004). In these three cases, like in ours, the coronary sinus was extremely dilated. Additional diagnostics could help to verify the diagnosis like echocardiography or magnetic resonance imaging. There are a number of possible drainage systems of the persistent left superior vena cava, e.g. via the coronary sinus (92%) or the left atrium (8%). In the majority of all cases (82 - 90%) a right-sided left superior vena cava is also present, or a persistent bridging vein (left brachiocephalic vein, 25 - 35% of cases). Other configurations are possible, with the left superior intercostal vein forming a communication between the left superior vena cava and the accessory hemiazygous vein forming a left sided azygous arch (Pretorius and Gleeson 2004).

## Conclusion

We reported a rare variant of the superior vena cava system with a persistent left superior vena cava and absence of a right vena cava superior that has been detected after catheter insertion with following CT. Early and adequate diagnosis is necessary for improved treatment with e.g. catheter-based systems.

## Patient’s consent

Written informed consent was obtained from the patient for publication of this case report and any accompanying images. A copy of the written consent is available for review by the Editor of this journal.

## Abbreviations

CT: Computer-assisted tomography
CVC: Central venous catheter.
